# Trigger and Substrate Mapping and Ablation for Ventricular Fibrillation in the Structurally Normal Heart

**DOI:** 10.3390/jcdd10050200

**Published:** 2023-05-02

**Authors:** Simon Christie, Sami Idris, Richard G. Bennett, Marc W. Deyell, Thomas Roston, Zachary Laksman

**Affiliations:** Faculty of Medicine, Division of Cardiology, University of British Columbia, Gordon & Leslie Diamond Health Care Centre, 2775 Laurel St., 9th Floor, Vancouver, BC V5Z 1M9, Canada

**Keywords:** J-wave syndromes, Brugada syndrome, early-repolarization syndrome, short-coupled VF, idiopathic VF

## Abstract

Sudden cardiac death (SCD) represents approximately 50% of all cardiovascular mortality in the United States. The majority of SCD occurs in individuals with structural heart disease; however, around 5% of individuals have no identifiable cause on autopsy. This proportion is even higher in those <40 years old, where SCD is particularly devastating. Ventricular fibrillation (VF) is often the terminal rhythm leading to SCD. Catheter ablation for VF has emerged as an effective tool to alter the natural history of this disease among high-risk individuals. Important advances have been made in the identification of several mechanisms involved in the initiation and maintenance of VF. Targeting the triggers of VF as well as the underlying substrate that perpetuates these lethal arrhythmias has the potential to eliminate further episodes. Although important gaps remain in our understanding of VF, catheter ablation has become an important option for individuals with refractory arrhythmias. This review outlines a contemporary approach to the mapping and ablation of VF in the structurally normal heart, specifically focusing on the following major conditions: idiopathic ventricular fibrillation, short-coupled ventricular fibrillation, and the J-wave syndromes—Brugada syndrome and early-repolarization syndrome.

## 1. Introduction/Background

Ventricular fibrillation (VF) is often the terminal rhythm leading to sudden cardiac death (SCD). SCD represents approximately 50% of all cardiovascular mortality in the United States, with the majority occurring in those with structural heart disease [1]. However, around 5% of individuals have no identifiable cause on autopsy [2,3]. Systematic evaluation of survivors with unexplained sudden cardiac arrest (SCA) can identify an etiology in around half of cases [4,5]. Insertion of an implantable cardioverter defibrillator (ICD) has a Class 1 indication in patients that survive SCA with an irreversible cause [6,7]. However, while ICDs save lives in this population, recurrent episodes of VF are associated with increased mortality and a substantial decline in quality of life [8]. Antiarrhythmic medications are effective at reducing ventricular arrhythmias (VAs) [9], but some individuals experience refractory disease despite multiple medications. Catheter ablation for VF has emerged as an effective tool to alter the natural history of this disease in individuals with refractory arrhythmias.

This review provides an overview of a modern approach to mapping and ablation of VF in the structurally normal heart, with a detailed review of 4 specific conditions, namely idiopathic ventricular fibrillation (IVF), short-coupled ventricular fibrillation (SCVF), and the J-wave syndromes (JWS)—Brugada syndrome (BrS) and early-repolarization syndrome (ERS). Many of the mechanisms of initiation and maintenance of VF as well as therapeutic techniques are shared between those with structural heart disease and those with apparently normal hearts. Additional entities, such as malignant mitral valve prolapse syndrome, which may only have a minor structural abnormality and which are increasingly recognized as a cause of ventricular arrhythmias, are beyond the scope of this paper.

## 2. Definitions and Epidemiology

While SCD in the structurally normal heart represents only 5% of all SCD, it is much more common in individuals <40 years old, where it is responsible for up to 30% of unexplained SCD [3]. Over the past several decades, several previously unknown disease entities have been discovered, which represent a substantial proportion of what was once considered to be IVF, including BrS, catecholaminergic polymorphic ventricular tachycardia (CPVT), Short QT syndrome, ERS, and SCVF. 

BrS has been widely recognized since 1992 and is present in 1 in 2000 individuals worldwide, with a higher prevalence in Southeast Asia [10,11]. Individuals with high-risk features of BrS, including a family history of SCD, personal history of syncope, or aborted SCD, are at significantly elevated risk of future VA [12]. The diagnostic criteria for BrS include the spontaneous or pharmacologically induced presence of ≥2 mm of coved ST elevation in the high-right precordial leads [13].

Early repolarization pattern (ERP) is a common finding, with a wide range in estimated prevalence, from 1–33% of the general population, depending on the exact definition used [14,15]. Early repolarization was initially described nearly a century ago and was considered a benign entity for the majority of this period [16,17]. However, ERP is present in a higher proportion of individuals with unexplained SCD [18,19,20], and when present in cohorts with established arrhythmic disorders such as BrS or CPVT, it portends a worse prognosis [21,22,23]. Additionally, specific morphologic aspects of the J-wave as well as localization to the inferolateral leads are associated with a higher risk for SCD [24]. ERS is diagnosed in individuals with the presence of (ERP) on the ECG with a history of resuscitated cardiac arrest or SCD without another identifiable cause [13].

IVF is a diagnosis of exclusion and is reserved for individuals with no identifiable cause for cardiac arrest following comprehensive investigation [13]. The true prevalence of IVF is challenging to estimate due to the non-uniformity in historical testing but has been reported in 1.2–6.8% of all SCD [5,25]. IVF represents a larger proportion of SCD in the structurally normal heart, where 45–60% of cases remain without another diagnosis [4,5]. Individuals with IVF are at high risk for recurrent ventricular arrhythmias (VA) requiring ICD therapy [26,27].

**Figure 1:**
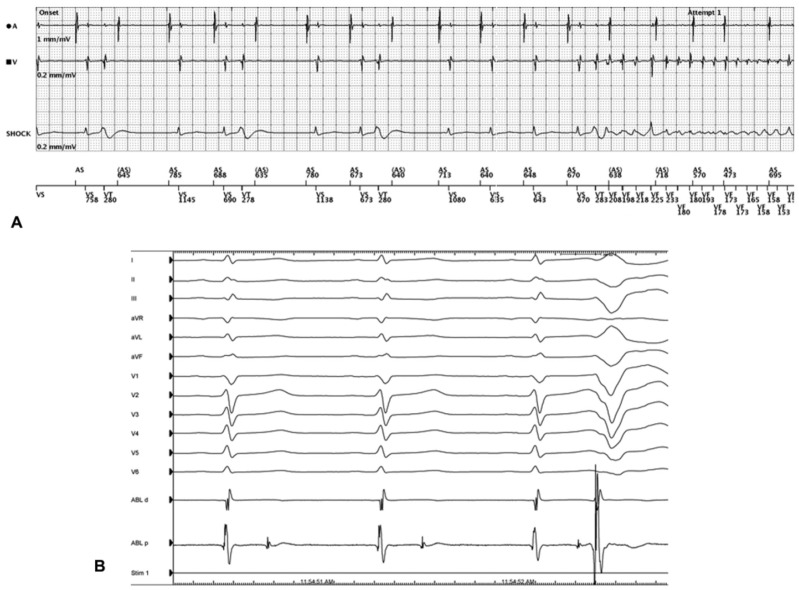
Short-coupled VF (Mellor 2016 [34], reproduced with permission). (**A**) Dual-chamber ICD electrogram demonstrating multiple short-coupled PVCs eventually triggering VF. (**B**) 12-lead surface ECG and intracardiac recordings from an ablation catheter positioned at the site of earliest activation. Following each sinus beat, there is a discrete local potential seen on the proximal pole of the ablation catheter representing localized depolarization in the distal Purkinje system which occasionally captures the surrounding tissue resulting in a PVC.

SCVF has been defined as unexplained VF that is initiated by a premature ventricular complex (PVC) with a coupling interval of <350 ms (Figure 1) [28]. SCVF has been recognized since the 1990s [29]. In the CASPER registry, SCVF accounts for 6.6% of unexplained cardiac arrest [28]. As SCVF is diagnosed almost exclusively in survivors with recurrent arrhythmia captured on monitoring devices, the actual incidence is likely to be higher. The rationale for classifying SCVF as a distinct entity separate from IVF derives from the work by Belhassen and colleagues demonstrating the efficacy of quinidine at preventing further VA in this population [28,30,31]. In those with refractory VA, catheter ablation has been shown to be an effective alternative [32,33]. 

## 3. Mechanisms of Ventricular Fibrillation

VF can occur as a result of destabilization of ventricular tachycardia (VT) or as a primary entity. It can be characterized into four phases: initiation, transition, maintenance, and evolution [35]. The initiation phase of primary VF is believed to be triggered by PVCs that fall in the “vulnerable window”, a time when the heart is particularly susceptible to malignant arrhythmias. The majority of these PVCs originate in the Purkinje system but can occur from anywhere in the ventricles [35,36]. Once triggered, one or more wavefronts move through the heterogeneously depolarized myocardium, leading to functional re-entry. The two main theories for the maintenance of VF are the multiple-wavefront hypothesis and the mother-rotor hypothesis [37]. The ‘multiple-wavelet hypothesis’ was first put forth by Moe et al., describing atrial fibrillation [38], and this same mechanism was proposed to underlie VF, with experimental data demonstrating steep cardiac restitution curves as a primary component to the wave-break phenomenon responsible for the multi-wavefront mechanism [39]. The mother-rotor theory proposes periodic rotors that serve to activate surrounding tissue with rapid frequency; it was demonstrated both experimentally as well as with computer simulation [40]. These two mechanisms often co-exist to perpetuate VF [37].

## 4. Catheter Ablation of VF

Several investigators have pioneered techniques for identifying and eliminating sources of arrhythmia in patients with VF and structurally normal hearts [41,42,43]. Careful evaluation utilizing both invasive and non-invasive methods can identify these causes in many individuals.

VF ablation can be separated into 2 main approaches: trigger ablation and substrate ablation. Trigger ablation focuses on identifying and eliminating PVCs that initiate VF, whereas substrate ablation relies on identifying abnormal areas of myocardium that function to propagate and sustain VF. Abnormal areas of delayed electrical activity within the epicardium or endocardium, as well as the Purkinje network itself, can function to sustain VF [33,44]. Elimination or modulation of these areas with radiofrequency (RF) ablation has been shown to be an effective therapy in highly symptomatic patients [33,41,42,43,44,45,46].

High-density endocardial and epicardial mapping with multipolar catheters is utilized to identify abnormal areas of activation. Endocardial mapping is performed by placing catheters on either side of the interventricular septum, with the LV accessed via atrial septal puncture or the retrograde aortic approach [36]. The epicardial space is typically accessed via a subxiphoid approach under fluoroscopic guidance. Areas are mapped first in sinus rhythm, then after induction of VA. Mapping after extra stimuli can also reveal areas of abnormalities concealed or masked during native conduction. Abnormal electrograms (EGMs) are required to meet one of multiple criteria: (1) low voltage (<1 mV), (2) split or fractionated EGMs with multiple potentials with at least two distinct components separated by >20 ms, (3) duration of >80 ms or late potentials extending beyond the QRS duration [43].

ECG imaging (ECGI) is a non-invasive tool that has been used to provide detailed epicardial activation mapping to guide ablation. The dominant commercially available ECGI system uses several hundred electrodes embedded in a wearable vest that are paired with a CT heart to provide a detailed 3D map of depolarization and repolarization. ECGI has been effective at identifying rotors and abnormal areas of electrical activity that have been shown to correlate with abnormal signals obtained via direct catheter mapping [44,47]. Ablation of these sites has proven effective at eliminating further VA [44]. 

### 4.1. Rotor Ablation

**Figure 2:**
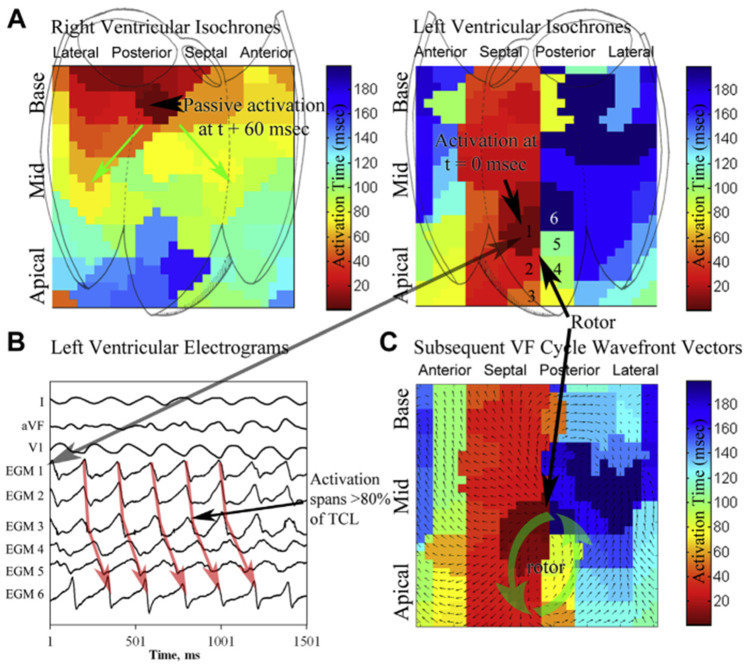
Biventricular VF Analysis with rotors (Krummen 2014 [47], reproduced with permission). (**A**) Isochronal analysis of the RV and LV during VF. LV isochrones show a rotor (cycle length 220 ms) in the septal LV. This rotor persisted for 15 continuous rotations (depicted ~5 s into VF); rotor activity was seen in 82% of all VF cycles in this patient. (**B**) Basket electrograms during VF, numbered (1 to 6) near the rotor core. Note that activation spans >80% of the VF cycle length. (**C**) Wave front vector analysis of the subsequent VF cycle, showing consistent rotation about the core with radial activation of distant tissue.

Rotors are defined as regions of rotational activity that control surrounding tissue activation (Figure 2) [48]. They often coincide with areas with abnormal electrograms [33,49]. Rotor mapping and ablation were first studied in canines by Krummen et al. [50]. Using 64-electrode basket catheters placed in both right and left ventricles, VF was induced and mapped in 9 dogs. Endocardial rotor sites were identified using phase analysis [51,52], then ablated with RF. After an average of 8 minutes of burn time, VF was rendered non-inducible in 6 of 9 dogs, while the remaining 3 dogs demonstrated significantly higher induction thresholds. 

In a proof-of-concept, the same procedure was then performed on a patient with ischemic cardiomyopathy and refractory VF with recurrent shocks [49]. Four unique rotor sites were identified that localized to border-zone tissue. Ablation was performed with an average of 6.3 minutes per site and area of 1.5–2 cm^2^ using an 8-mm ablation catheter at 55 watts and 55 °C. Sham ablation was performed >3 cm from any identified rotor site with no effect on the VF. After ablation of the rotors, VF was non-inducible, and the patient remained free from ICD therapies after 1 year without the use of antiarrhythmic medications.

Krummen and colleagues later demonstrated the importance of rotors in the stability of early VF in 26 patients undergoing ablation for recurrent VA [48]. The studies were done under general anesthesia and mechanical ventilation. A decapolar catheter was placed in the coronary sinus, and a quadripolar catheter placed in the RV. Two 64-electrode basket catheters were used, with 1 in each ventricle. They found rotors in 16 of 19 patients with VF and all patients with sustained VF. Individuals with sustained VF had greater rotor stability as well as a greater number of maximum rotations around the rotor compared to individuals without sustained VF. In 7 patients in whom VF was induced twice, the location of the rotors remained consistent. Rotors lasting >45% of all VF cycles differentiated those with sustained versus non-sustained VF, with a sensitivity of 100% and specificity of 93%.

While the studies by Krummen et al. describe rotors in individuals with structural heart disease, the principle of rotors functioning to sustain VF also applies to those with apparently normal hearts [43,46]. Their work offers excellent insight into the mechanics of VF initiation and stability.

### 4.2. J Wave Syndromes 

Brugada syndrome (BrS) and early-repolarization syndrome (ERS) have been categorized as J-Wave syndromes due to their appearance on the surface ECG. There remains debate as to whether the electrographic phenomenon is primarily due to repolarization versus depolarization, with both seeming to have the capacity for such an appearance [53,54,55]. Individuals with ERS and BrS with recurrent episodes of VF have both been treated successfully with ablation.

### 4.3. Brugada Syndrome

Haissaguerre and colleagues were the first group to demonstrate the efficacy of RF ablation for the treatment of recurrent VT and VF in BrS [46]. In this small proof-of-concept study, three individuals with BrS and frequent episodes of VF with frequent PVCs were enrolled in electrophysiology study (EPS). The earliest site of activation of the PVCs was determined using endocardial mapping, with all sites located in the right ventricle (two in the right ventricular outflow tract (RVOT) and one in the anterior Purkinje network). The sites were ablated with 7 to 10 minutes of RF application. After 17 months of follow up no patients had recurrent VA. 

**Figure 3:**
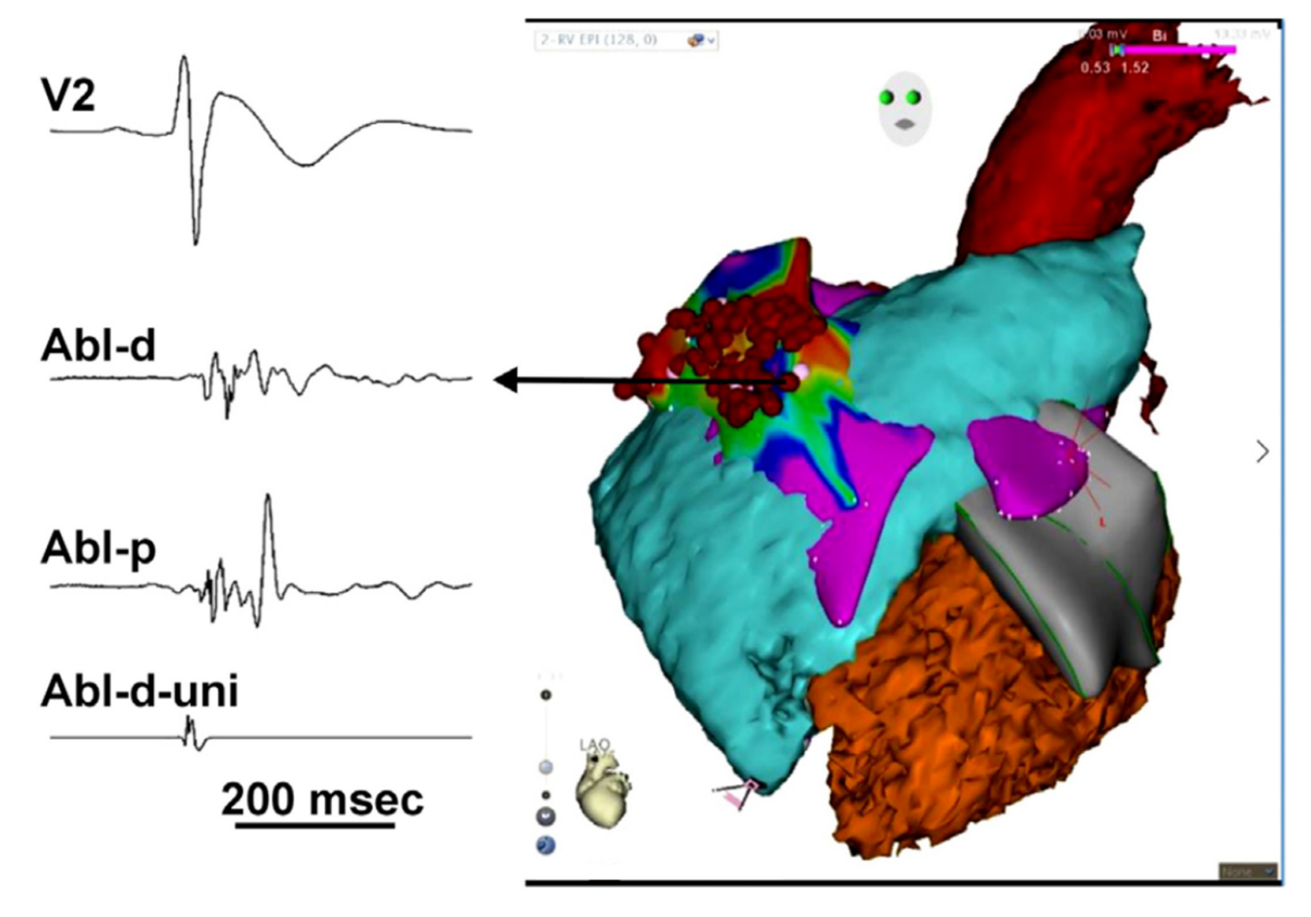
3D anatomical mapping in BrS. (Nademanee 2017 [62], reproduced with permission). The CARTO-Merge map shows a left anterior oblique view of the cardiac CT scan that is merged with the electro-anatomic maps of the right ventricular outflow tract epicardium of a patient with BrS with frequent ICD discharges. The arrow shows abnormal prolonged low-voltage fractionated electrograms recorded from the site of the ventricular outflow tract epicardium. Abl-d, bipolar ablation distal; Abl-p, bipolar ablation proximal; Abl-d-Uni, unipolar ablation distal.

One challenge with trigger ablation is the dependency on frequent ectopy to accurately map the region of interest. PVCs in the BrS population are rare and, if present, occur mostly during sleep [56]. It was discovered that patients with BrS have prolonged and fractionated propagation of depolarization in the RVOT [57]. Nademanee and colleagues published the first study focused on epicardial substrate mapping and ablation in 9 patients with BrS and frequent episodes of VF [43]. All patients underwent EPS with both endocardial and epicardial mapping. Electrical mapping was integrated with a CT image done within 24 h of the procedure. The CARTO system [58] was used to create maps of both signal duration and amplitude. All 9 individuals had abnormal substrate identified exclusively in the anterior aspect of the RVOT epicardium (Figure 3). These areas were later shown to correlate with increased collagen deposition and decreased connexin-43 expression [59]. Ablation of these sites eliminated further VF and normalized the Brugada ECG pattern in 8 out of 9 patients, with the last patient having a substantial decrease in the number of ventricular events. Importantly, abnormal potentials noted during epicardial mapping were not seen during endocardial mapping alone; thus, without an epicardial approach, these areas harboring lethal arrhythmic substrate would not have been found or ablated. This finding has been corroborated in other studies [60,61]. 

Brugada et al. later published their results of 14 patients with BrS with recurrent VA [63]. They performed detailed epicardial mapping and ablation, augmented with the use of flecainide during the procedure. They identified low-voltage and abnormal EGMs in the RVOT as well as the anterior RV free wall, which increased markedly from 17.6 cm^2^ to 28.5 cm^2^ after flecainide infusion. After ablation of these areas, the type 1 Brugada pattern had resolved, and VT/VF was non-inducible. After a median follow-up of 5 months, the type 1 ECG pattern remained absent, even after flecainide provocation testing. It is important to reiterate that the identification of abnormal areas of electrical activity in BrS is substantially increased with Class I antiarrhythmic provocation, as Nademanee and colleagues reported that, prior to regular use of ajmaline provocation during procedures, some patients would have return of the type 1 Brugada ECG pattern, which correlated to future episodes of VA [62].

Pappone and colleagues subsequently reported on their experience with mapping and ablation of 191 patients with BrS, identified from their ICD registry [64]. The cohort consisted of 2 groups: group one included 88 patients with a prior history of cardiac arrest or syncope secondary to VT/VF, and group two had no history of VA. Regardless of the clinical presentation, individuals with inducible VT/VF had larger areas of abnormal electrical potentials. An abnormal substrate area of >4 cm^2^ identified patients with inducible arrhythmias with an area-under-the-curve of 0.98. Patients with inducible VT/VF had an average abnormal area of 8 cm^2^ compared to just 1 cm^2^ in the non-inducible group. Ajmaline infusion substantially increased the size of baseline abnormal areas, with a concomitant rise in the number of patients with inducible VT/VF. Ablation of these areas prevented further VA.

One of the challenges of epicardial ablation in BrS is the proximity of the coronary arteries to areas of abnormal late potentials. Kamakura et al. attempted endocardial ablation in 16 patients with abnormal epicardial potentials in close proximity to the right coronary artery, prohibiting an epicardial approach [65]. Endocardial ablation was successful in 53.3% of sites, with a decrease in VF burden from a median number of 7 episodes pre-ablation to 0 episodes over 25 months follow-up. While this approach is not ideal, it may serve as an additional option when epicardial ablation is not possible.

A recent systematic review of ablation in BrS found that in 388 patients, VF was non-inducible in 87.1% of patients after ablation, and resolution of type 1 ECG was seen in 74.5% [66]. Recurrent VA was seen in 17.6%. Use of Class I antiarrhythmics to augment mapping was only used in 80% of cases, and as noted earlier, residual substrate was a marker for recurrence. Thus, with the routine use of Class I antiarrhythmics during substrate mapping, it is likely that results would be even better. 

### 4.4. Early-Repolarization Syndrome

Nademanee et al. published one of the largest multicenter studies to date, evaluating the role of ablation in ERS and BrS [44]. Fifty-two patients with ERS (33 patients with ERS and Brugada ECG pattern, and 19 patients with ERS without Brugada pattern) from 6 different sites were taken for mapping and ablation. Two distinct phenotypes were identified: the first group of 40 patients had late-depolarization abnormalities in the RV epicardium, whereas 12 patients had no such late-potentials identified. The RVOT and inferior RV epicardium were the 2 predominant locations of these late potentials in the first group. Whereas the second group had no identifiable abnormal substrate but were found to have PVCs originating within the Purkinje network. A mix of substrate and trigger ablation was performed on 43 of 52 patients, with a 91% success rate of no VF recurrence over a median of 31 months follow-up. ECGI was performed for patients in Thailand and France to map areas of early VF drivers. Areas of VF drivers colocalized with areas of low voltage late potential EGMs on the epicardial surface with 100% correlation. Among the individuals without abnormal depolarization on mapping (group two), VF drivers were located predominately in the inferior ventricular wall—septum 100%, LV 100%, RV 60%—suggesting a possible mechanism of Purkinje re-entry being the predominant early driver of VF.

Boukens et al. reported on the possible mechanism for the early repolarization pattern encountered in a patient with ERS and recurrent VA with the early repolarization seen in the inferior leads [67]. No clear trigger could be identified for ablation; thus, the patient underwent open thoracotomy epicardial mapping and ablation, which was felt to be required due to prior chest surgery as a child that deterred electrophysiologists from a subxiphoid epicardial approach. They identified an area with low voltage and delayed depolarization on the inferior side of the RV free wall. Detailed mapping identified local depolarization in this area coinciding with the surface ECG J-wave. Biopsied tissue from this site revealed extensive fibrosis with a few myocytes traversing the area, akin to what is seen after myocardial infarction. Ablation at this site eliminated the early repolarization ECG pattern as well as further episodes of VF.

### 4.5. Idiopathic Ventricular Fibrillation/Short-Coupled Ventricular Fibrillation

Haissaguerre and colleagues were the first to systematically study the role of ablation in patients with idiopathic VF, with no phenotypic evidence of BrS or ER [41]. They identified 27 patients without known heart disease who were resuscitated after cardiac arrest. These patients had recurrent episodes of VF despite an average of 3.6 antiarrhythmic drugs. All cardiac testing, including echocardiography, coronary angiography, exercise stress testing, and sodium channel blockade provocation testing, was normal; however, no patients underwent cardiac MRI. The common findings among all patients were early-coupled PVCs with an average coupling interval (CI) of 297 ms, frequently seen in the post-resuscitation period. These PVCs demonstrated an identical morphology to the PVC that triggered VF. The average number of PVCs prior to ablation were 7841 per 24 h. 

The triggers were mapped by identifying the earliest electrogram relative to the onset of the ectopic QRS. Premature beats preceded by brief (10 ms), sharp deflections by <15 ms were thought to represent distal Purkinje potentials, whereas the same sharp deflections that had a longer latency time before the QRS were thought to represent proximal Purkinje potentials, as described previously [68]. The absence of any preceding sharp deflections represented ventricular myocardial origin of the ectopic beat. The earliest site of activation was targeted for ablation. 

Twenty-four patients had spontaneous PVCs. The PVCs originated from the RVOT in 4 patients, whereas the other 20 patients had PVCs originating from the Purkinje network: 7 from the right, 9 from the left, and 4 from both ventricles. Repeated Purkinje potentials were recorded prior to the PVCs, occasionally with varying coupling intervals and QRS morphology, suggesting differing activation patterns despite the same site of origin. The remaining 3 patients with no PVCs during EPS were felt to have ectopic foci originating from the Purkinje network based on morphology recorded on their prior 12-lead ECGs, with the sites subsequently localized using pace mapping.

After ablation of these sites, 24 patients (89%) demonstrated a substantial decrease in the burden of PVCs, to an average of 28 per 24 h, with no documented VF, SCD, or syncope after an average of 24 months of follow-up. Three patients had late recurrence of PVCs with the same morphology, and all 3 eventually developed recurrent VA. 

In a long-term follow up study that included an additional 11 patients with IVF initiated by short-coupled PVCs, 7 out of 38 patients had recurrent VF at an average of 24 months after initial ablation [69]. Five out of 7 patients underwent a second ablation procedure, with complete resolution of further VF episodes. The only predictive feature of recurrent VF was the occurrence of transient bundle-branch block from the originating ventricle during the ablation procedure (known as “bumping” phenomenon), likely due to inability to accurately map the source of the ectopy. This study was pivotal for proving the efficacy of targeting triggers for preventing lethal arrhythmias in IVF. Ablation reduced the number of VT/VF episodes from a median of 4 to 0, with 36 of 38 patients free from VF after 52 months of follow-up. Only 13% of patients were on antiarrhythmic medications after ablation.

PVCs originating from the RVOT in structurally normal hearts are most often considered to be benign, with excellent long-term prognosis [70,71]. However, there is a small subset of patients at increased risk of SCD caused by RVOT PVCs [72]. Noda and colleagues reported on 101 patients in whom RF ablation was performed for recurrent VA triggered by PVCs originating from the RVOT. Among these patients, 5 had spontaneous VF, and another 11 had a history of syncope. Ablation at the earliest site of activation eliminated further episodes of VA in all patients after an average of 54 months follow-up. The clinical factors differentiating those at high risk of SCD and those with benign PVCs can be challenging to identify. The authors found no difference in the age, sex, family history, burden of PVCs, or coupling intervals between those with hemodynamically stable RVOT-VT and those with polymorphic VT/VF. The only predictive factor was a history of syncope in the group with polymorphic VT/VF; thus, individuals with any history of unexplained syncope in the context of RVOT PVCs should be highly considered for ablation.

While most of the studies to date for IVF ablation have focused on Purkinje or RVOT triggers, Van Herendael et al. identified papillary sites among triggers in a small cohort in 2014 [73]. Papillary muscles are well known sites for idiopathic PVCs and can be challenging sites to ablate. The fact that Van Herendael et al. employed intracardiac ultrasound for a substantial number of patients is intriguing, as this was cited as a key factor in accurately identifying the papillary muscles as the sites of origin for the triggering PVCs. However, one caveat is the distinction between Purkinje-mediated and papillary-muscle-originating ectopy may not be black and white, given reports of Purkinje fibers extending into the papillary muscles [74].

### 4.6. Purkinje Network Modification

Complete elimination of the trigger has been the end-point for many of the pivotal studies, but Purkinje network modification at sites other than the earliest activation site may be sufficient to eliminate recurrent episodes of VF [42,45]. The Purkinje system is thought to play an important role in both triggering ectopy as well as sustaining early fibrillation with re-entrant circuits within the Purkinje network [75]. Nogami et al. report a case of a 54-year-old man who had early coupled PVCs initiating VF, with Purkinje potentials recorded prior to the QRS [42]. The earliest site was felt to be quite proximal and could not accurately be determined; thus, they opted to ablate a more distal site in the Purkinje network. VF was non-inducible after ablation, and after 14 years of follow-up the patient had no further episodes of VF. The belief was by creating intra-Purkinje block; early VF was no longer able to sustain itself within the Purkinje network. In a cohort of 54 patients with structural heart disease, Haissaguerre and colleagues demonstrated further evidence for the Purkinje network’s capacity to function as a primary driver of early VF [76].

### 4.7. Limitations of Catheter Ablation 

The data included in this review represent experience described by highly specialized centers and thus may not represent all individuals within each diagnostic category. Given the rarity of these conditions, there are no randomized clinical trials, and thus some caution must be given to the interpretation of the results. There remain significant gaps in our understanding of SCD, not least our ability to identify those at greatest risk. SCD is a complex combination of various risk factors that evolve over time. Accurate prediction of individuals most at risk of sudden death, and therefore most likely to benefit from intervention, challenging [77]. 

A major limitation to trigger ablation of VF is the reliance on spontaneous PVCs for accurate mapping. In individuals with a low burden of PVCs, antiarrhythmic medications and procedural sedation can further decrease the likelihood of PVCs during the procedure. In addition, in those with multiple PVC morphologies it may not be clear which ones can trigger VF. One method to circumvent low-frequency PVCs is pace-mapping; however, this method does not guarantee that the source of PVC is eliminated even when the morphology is closely matched. When no trigger or abnormal substrate is identified, it is not clear what endpoint should be targeted. 

Another drawback of catheter ablation in the setting of VF is hemodynamic instability, particularly in the acute setting. Many individuals presenting in electrical storm have had multiple shocks, which can cause myocardial stunning, increasing the potential for refractory VF with catheter manipulation. It is essential to have an experienced team, with the availability of invasive hemodynamic support, in the event of circulatory collapse; therefore, these procedures should only be performed in specialized centers.

Anatomic considerations, including the proximity of the coronary arteries to sites of abnormal myocardial substrate as well as sites adjacent to the AV node and His bundle, limit the ability to safely ablate in these areas.

Lastly, while these studies have proven the efficacy of ablation in reducing VA, a minority of patients will experience recurrent events; thus, ablation is not a substitute for ICD implantation.

## 5. Future Directions

As the use of wearable technology and implantable loop recorders increases, the amount of data available for analysis will markedly increase. Combined with rapidly advancing artificial intelligence (AI), we are likely to see significant improvement in our overall understanding of VF predictors, mechanisms, and disease progression. AI has already proven effective at identifying critical areas for ablation [78]. Technological advances, including invasive and non-invasive mapping as well as novel ablation systems such as pulsed field ablation or stereotactic external beam radiation, will likely further improve the efficacy and safety of ablation. 

Given the rarity of many of these conditions, there is a need for large multinational prospective databases and, ideally, randomized clinical data comparing ablation strategies and medical approaches to guide intervention.

## 6. Conclusions

**Figure 4:**
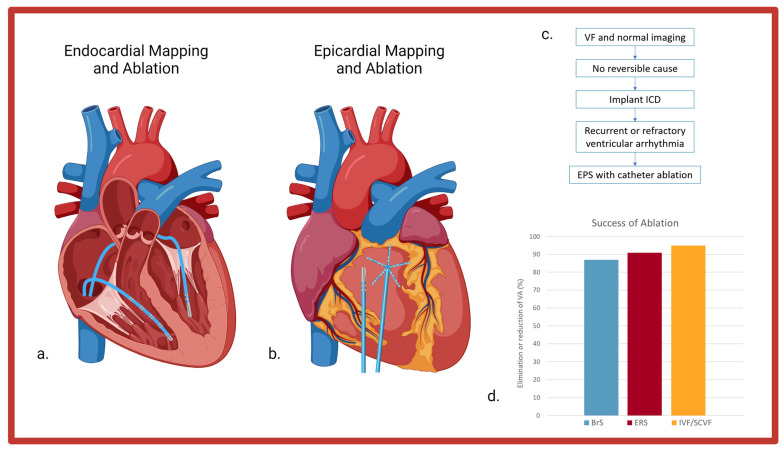
(**a**). Decapolar mapping catheter and ablation catheter via an endocardial approach. (**b**). Multipolar mapping catheter and ablation catheter via an epicardial approach. (**c**). Simplified step-wise approach to individuals with ventricular fibrillation and a structurally normal heart. (**d**). Success rates of VF ablation in different diagnostic categories. VF = ventricular fibrillation. ICD = implantable cardioverter defibrillator. EPS = electrophysiology study. BrS = Brugada Syndrome. ERS = early repolarization syndrome. IVF = idiopathic ventricular fibrillation. SCVF = short-coupled ventricular fibrillation. VA = ventricular arrhythmia.

Catheter ablation of VF in individuals with primary arrhythmic syndromes is an effective therapy for reducing recurrent VA (Figure 4). VF in structural heart disease may also be amenable to ablation; however, this was beyond the scope of the present review. As our understanding of the mechanisms of initiation and perpetuation of VF increases, so does our capacity to intervene and potentially cure these conditions. Both trigger and substrate ablation have been shown to be effective at either eliminating or drastically reducing the number of arrhythmic episodes in highly symptomatic individuals. 

## Data Availability

Not applicable.
